# Multiple anti-seizure medications use and pattern of seizure control in children with epilepsy at neurology follow up clinic, Tikur Anbessa specialized hospital, Addis Ababa, Ethiopia

**DOI:** 10.4314/ahs.v23i2.84

**Published:** 2023-06

**Authors:** Deginet Endayen, Moges Ayalew, Mengistu Getahun

**Affiliations:** 1 St Paul's Hospital Millennium Medical College; 2 Addis Ababa University; 3 Addis Ababa University College of Health Sciences

**Keywords:** Multiple antiseizure medications, seizure-control, children, Ethiopia

## Abstract

**Back ground:**

Antiseizure drug treatment is the mainstay of the management of epilepsies. Thirty percent of individuals with epilepsy experience refractory or drug resistant seizures which often require treatment with combinations of antiseizure drugs.

**Methods:**

This was a cross sectional descriptive study of three hundred fifty-five children with epilepsy attending Tikur Anbessa hospital in Addis Ababa, Ethiopia. Children with epilepsy who had been on Antiseizure medications for six months and more were consecutively enrolled from October 1 2018 to December 30 2018 to reach the calculated sample size. The history and laboratory result information were extracted from patient's records and was supplemented by direct inquiry. Data was analyzed using SPSS for windows version 24. Multivariate logistic regression analysis was done for those that had P-value (<0.05) on bivariate analysis and adjusted odds ratio were used to explore the association.

**Results:**

One hundred twenty nine of the 355 children (36.3 %) were on multiple AEDs; 114(32.1%) were on dual therapy while 15(4.1%) were on three anti epileptic drugs.

**Conclusion:**

One third of children with epilepsy attending the pediatric neurology clinics were being managed with multiple Anti-seizure drug therapy. Almost half of the participants had achieved seizure freedom in the past six months.

## Introduction

Epilepsy is the commonest neurological condition affecting people of all ages, race and social class. There are an estimated 50 million people with epilepsy in the world of whom up to 75% live in resource poor countries with little or no access to medical services or treatment. 1

The problem is particularly compounded in areas where patients with epilepsy lack access to neurologists, or even to physicians, and where diagnostic methods such as neuro imaging and electroencephalography (EEG) are not available. 2

In low- and middle-income countries, about three fourths of people with epilepsy may not receive the treatment they need because of <50%availability of generic antiseizure medications in the public sector. 3 The reported size of the epilepsy treatment gap in sub-Saharan Africa varies widely, ranging from 23% in Senegal to 100% in Uganda, Tanzania, Gambia, and Togo. 4 The major contributing factors for the treatment gap are lack of primary health workers trained to diagnose and treat epilepsy, limited access to health facilities particularly in rural areas, social stigma, misinformation, and traditional beliefs, limited opportunities for training in neurology and lack of diagnostic modalities like EEG and MRI. 3,4

With effective drug treatment, up to 70% of individuals with active epilepsy have the potential to become seizure free and go into long-term remission shortly after starting drug therapy and that around 70% of individuals can achieve seizure freedom using a single antiepileptic drug. 5. The remaining 30% of individuals experience refractory or drug resistant seizures which often require treatment with combinations of antiepileptic drugs or alternative treatments such as epilepsy surgery. 5,6,7,8

Use of multiple Antiseizure medications (ASMs) has been reported high. Studies showed up to one quarter of people with epilepsy take two or more antiseizure medications and this proportion has been reported to increase to over 75% among medically refractory patients attending tertiary referral centres. Additionally, there is a high probability of being co-prescribed with other drugs at some point in life. 9,10

The choice of optimal polytherapy poses difficulty for several reasons. First and foremost, there are limited data regarding favourable or unfavourable combinations. With few exceptions, there is little systematic evidence that any two combinations are any more or less effective as any other. Polytherapy may also be associated with pharmacokinetic interactions and drug side effects. 11,12,13

One Indian study which was conducted in tertiary hospital in Bangalore from January 2011–March 2012concluded that selection of rational and safer Antiseizure medications treatment options, and regular monitoring for adverse effects play a crucial role in achieving seizure freedom and optimal quality of life in patients with epilepsy. In this study frequency of seizures and Antiseizure medications side effect was significantly higher among patients on polytherapy than among those on monotherapy. 14

Few hospital-based studies done in all age group showed, the use of multiple Antiseizure medications in epilepsy patients in Ethiopia is variable. 15,16,17. But there was no study done in specific pediatric age group regarding antiseizure medication use in Ethiopia. This study was conducted mainly in pediatric age group in one of the terticiary centre the contribution will be paramount.

The study done in University of Gondar referral hospital to assess drug therapy of epileptic seizures among adult epileptic patient showed the rate of polytherapy use was 19.3%. Phenobarbitone was found to be the most commonly prescribed antiseizure medication. 16.

In other study conducted to determine drug use evaluation of anti seizure medication use in all age group in Bishoftu general hospital Ethiopia showed the use of monotherapy and dual therapy was 78.6% and 21.4%. 15 In this study Phenobarbitone was the commonest monotherapy 98.2% followed by sodium valproate 1.8%.

The study from Eastern African country Uganda which was done to assess multiple anti-epileptic drug use in children with epilepsy in Mulago hospital concluded that one third of children with epilepsy attending the epilepsy clinics are being managed with multiple Anti-Epileptic drug therapy. 18 However, many of these children might have been inappropriately initiated onto multiple Antiseizure medications as they were on lower than recommended maintenance doses. 18

According to a tertiary teaching hospital Indian study, data from five hundred twenty-eight patients with epilepsy showed, 47% of cases are refractory for Antiseizure therapy. Polytherapy was most frequently used in all type of epileptic seizures. Sodium Valproate was the most frequently prescribed Antiseizure medications followed by Phenytoin and Carbamazepine. 19.

The objectives of this study were to assess multiple antiseizure medications use, pattern of seizure control and factors associated with it. There was no study conducted in specific pediatric age group regarding use of antiseizure medication use in Ethiopia. This study was conducted mainly in pediatric population; the contribution of the study will be paramount.

## Methods and Materials

Our study was conducted in the Pediatric Neurology follow-up clinic of Tikur Anbessa Specialized Hospital from October 1 to December 30 2018.

Tikur Anbessa Specialized Hospital was established in 1974 and is the teaching hospital of School of Medicine, School of Pharmacy, School of Public Health and School of Allied Health Sciences of Addis Ababa University. It is also the largest referral hospital in Ethiopia with over 700 beds and serves as a training centre for undergraduate and postgraduate medical students as well as other health providers.

The Pediatric Neurology Clinic serves a total of 500 to 700 children with various neurological diagnoses each month (clinical audit of pediatrics department 2009 E.C), and is open each working day It is run by pediatric neurologist, pediatric residents, general practitioners and nurses. Approximately 60 % of children seen in this clinic have a diagnosis of epilepsy.

We included all children 1 month through 18 years who were treated with antiseizure medication for 6 months or longer for epilepsy over a 3-month period, and whose parents or care givers consented to the study.

We excluded children who were actively convulsing during the clinic appointment.

389 study participants were recruited using a convenience sample over 3 months.

The data collection format was pre-tested prior to the actual data collection. Epilepsy details were abstracted from the charts and supplemented by direct inquiry at the time of clinic visit. Two trained nurses and one medical doctor (Pediatric resident) underwent a half day of training about the study and data collection, and then collected the data. Oversight was provided by the primary investigator to confirm that all information was being recorded and collected correctly. The collected data was checked for completeness, accuracy and clarity by the principal Investigator.

Data was entered and analysed using statistical package for social science (SPSS) for windows version 24. Frequency tables and charts were used to present results of categorical variables. Multivariate logistic regression analysis was done for those that had significant P-value (<0.05) on bivariate analysis and adjusted odds ratio were used to explore the association.

This research was approved by the Research Ethics Committee of Department of Neurology, Department of Pediatric and Child Health and Institutional Review Board of the College of Health Science of Addis Ababa University.

Epilepsy was defined as the presence two or more unprovoked seizure separated by >24 hour. 20. Seizure control was dichotomized into two groups: Controlled, defined as no seizures in the preceding 6 months, versus Uncontrolled, defined as ongoing seizures in the preceding six months.

Multiple antiseizure medication Use was defined as the use of two and or more antiseizure medications for the treatment of epilepsy. 21

## Results

From a total of 389 Epilepsy patients, 355 study participants were included in this study. 34 patients were excluded. Among excluded 34 patients; 19 patients were on antiseizure drugs for < 6 months, 14 patients were due to lack of parent or guardian consent and one patient had seizure during the follow up visit. The sociodemographic variables of the study participants are detailed in the Table 1.

**Table 1:** Demographic characteristic of participants for age < 18 years, in Tikur Anbessa Hospital, Pediatric neurology follow up clinic, 2018

Characteristics		Number	Percent
Age at time of visit in year.	< 1	5	1.4%
	1 – 5	117	33%
	5 – 10	129	36.3%
	10 - 18 year	104	29.3%
Sex.	Female	145	40.8%
	Male	210	59.2%
Caregiver.	Parent	312	87.2%
	Relative	36	10.1%
	Others	7	2%
Caregiver educational level.	No formal education	46	13%
	Primary level	107	30.1%
	Secondary level	120	33.8%
	Tertiary level	82	23.1%
Caregiver employment status.	unemployed	181	51%
	employed	174	49%

Males comprised 59.2% of participants. Participants represented a variety of ages, however only 1.4% were less than 1 year of age.

Most of participants were from Addis Ababa city (257; 72.4%). Seventy-one (20%) of participants were from Oromia region, (14; 3.4%) were from SNNRP region and (12; 3.4%) were from Amhara region. These regions are the main federal regions of Ethiopia with more than 80% rural population.

### Clinical Characteristic

Approximately two thirds of children had seizure onset between 1-10 years and about one third prior to age 1 year. The age of onset of seizure in 222 (63.1%) was between 1-10 year and the onset in 115 (32.4%) children was less than one year.

Two hundred seventy-seven (78%) children were on AEDs therapy for more than two years and 78 (22%) were on AEDs therapy for six months to two years. Generalized onset of seizure was diagnosed in 294 (82.8%) of children and focal onset was in 61 (17.2 %) of children. West syndrome was diagnosed in eleven children and Lennox Gastaut syndrome in two children. In this study Generalized onset of seizure was probably over diagnosed and epilepsy syndromes were under diagnosed. The above result might be resulted from more than 90 percent of patient with epilepsy in the clinic had been evaluated by pediatric residents in their first visit.

Seventy-one (20%) of children were diagnosed to have comorbid conditions. Among these children 35 (49.3%) of children had developmental delay and twenty (28.1%) children diagnosed to have cerebral palsy. Three children had language impairment, two children had visual impairment and three children had headache disorders. Eight children had psychological problems among these, three children had autistic spectrum disorder and five children had attention deficit hyperactivity disorder.

### Number of Antiseizure medications Used

**Figure 1:**
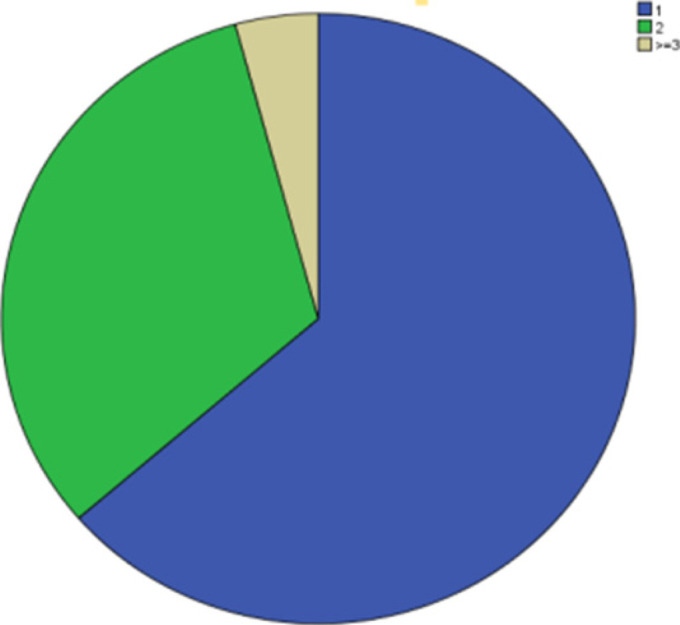
Number of Antiseizure medication (ASMs), in Tikur Anbessa Hospital, Pediatric neurology follow up clinic, 2018

One hundred twenty nine of the 355 children (36.3%) were on more than one Antiseizure medications; 114(32.1%) were on dual therapy while 15(4.1%) were on three Antiseizure medications. (Figure 1)

Of the226/355 participants on monotherapy, 76 (33.6 %) were on phenytoin, 71 (31.4%) were on phenobarbitone, 58 (25.6 %) were on sodium valproate and 20 (8.8%) were on carbamazepine. The lists of medications available in our country are Phenobarbitone, Phenytoin, Carbamazepine, Clonazepam and Sodium Valproate. Currently, lamotrigine is available only in few pharmacies and at very high prices making it unaffordable to nearly all of our patients.

Three hundred (84.5 %) of children were receiving correct maintenance dose (which is within the therapeutic range as put in most literatures) of Antiseizure medication and 28(7.5%) receiving below the recommended maintenance dose and twenty-seven (7.6%) children receive above recommended maintenance dose.

The most common drug combination was sodium valproate and phenobarbital, 46(40.4%) followed by phenytoin and phenobarbitone, 25 (21.9 %) and sodium valproate and clonazepam, 14 (12.4 %).

In this study Serum level measurement was particularly determined by automated enzyme-multiplied immunoassay technique in 46 (13.4%) of children who had poorly controlled seizure to assess compliance to antiseizure medication. Thirty-two (69.6%) of children serum level was in therapeutic range, 8 (17.4%) of children above therapeutic range and in 6 (13.6%) children below the range.

### Factors associated with multiple antiseizure medication therapy

On Univariate analysis children who had ongoing seizures in the past six months were two-fold more likely to use multiple Antiseizure medications as compared to children who were seizure free in the past six months. Children on monotherapy were more likely to have history of status epilepticus as compared to children with multiple Antiseizure medications use (p = 0.07). (Table 3)

**Table 3:** Univariate Logistic Regression of variables with Number of current Antiseizure medication (ASMs), in Tikur Anbessa Hospital, Pediatric neurology follows up clinic, 2018

Variables	Number of current ASMs				Univariate Analysis		
	>=2 ASMs		One ASMs		Odds Ratio	95% CI	p value
	n	%	n	%			
**Sex**							
Male	80	38.1	130	61.9	1.13	0.85, 1.50	0.411
Female	49	33.8	96	66.2	Ref		
**Age at seizure onset**							
< One year	44	38.3	71	61.7	Ref		
> One year of age	85	35.4	155	64.6	0.93	0.69, 1.23	0.599
**Duration of treatment**							
6 months - 2 years	22	28.2	56	71.8	0.73	0.50, 1.07	0.108
> 2 years	107	38.6	170	61.4	Ref		
**Type of Seizure**							
Generalized Onset	117	36.6	203	63.4	1.07	0.66, 1.72	0.793
Focal Onset	12	34.3	23	65.7	Ref		
**History of status epilepticus**							
Yes	7	15.2	39	84.8	0.39	0.19, 0.77	**0.007**
No	122	39.5	187	60.5	Ref		
**Other comorbid neurological condition**							
Yes	24	33.8	47	66.2	0.91	0.64, 1.31	0.625
No	105	37	179	63	Ref		
**Seizure control on treatment**							
No seizure in the past six month	39	23.6	126	76.4	Ref		
Seizure in the past six month	90	47.4	100	52.6	2.00	1.47, 2.74	**<0.001**

### Seizure control in children using anti-epileptic drug therapy

One hundred sixty-seven (47%) of children were seizure free in the past six months. Of those who were not seizure free, 91(25.6%) had monthly seizures, 59 (11%) had weekly seizure and 24(6.8%) had daily seizures.

Thirty-nine children on multi Antiseizure medications, 39/129 (30.2 %) had good seizure control compared to 126/226 (55.7 %) patients on monotherapy.

### Factors associated with seizure control

On both Univariate and multivariate analysis, children on multiple Antiseizure medications were less likely to attain seizure control (p=0.001) as compared to children on monotherapy. The presence of comorbid neurological conditions was also associated with poor seizure control (p=0.044). (Table 5 and table 6).

**Table 5:** Univariate Logistic Regression of variables with Seizure control on treatment, in Tikur Anbessa Hospital, Pediatric neurology follow up clinic, 2018

Variables	Seizure control on treatment				Univariate Analysis		
	Seizure in the past six month		No seizure in the past six month		COR	95% CI	p value
	n	%	n	%			
**Sex of the child**							
Male	113	53.8	97	46.2	1.01	0.83, 1.23	0.896
Female	77	53.1	68	46.9	Ref		
**Care giver of the child**							
Parent (mother or father)	169	54.2	143	45.8	1.15	0.80, 1.64	0.455
Relative of the child	17	47.2	19	52.8	Ref		
Others	4	57.1	3	42.9	1.21	0.58, 2.51	0.608
**Age of onset of seizure**							
< One year	65	56.5	50	43.5	Ref		
> One year of age	125	52.1	115	47.9	0.92	0.75, 1.13	0.425
**Duration of treatment**							
6 months - 2 years	42	53.8	36	46.2	1.01	0.80, 1.27	0.948
> 2 years	148	53.4	129	46.6	Ref		
**Patient had epilepsy syndrome**							
Yes	6	46.2	7	53.8	0.86	0.47, 1.56	0.614
No	184	53.8	158	46.2	Ref		
**Type of seizure**							
Generalized Onset	170	53.1	150	46.9	0.93	0.69, 1.26	0.639
Focal Onset	20	57.1	15	42.9	Ref		
**Patient had history of status epilepticus**							
Yes	26	56.5	20	43.5	1.06	0.81, 1.40	0.653
No	164	53.1	145	46.9	Ref		
**Patient had comorbid neurological condition.**							
Yes	45	63.4	26	36.6	1.24	1.01, 1.53	**0.044**
No	145	51.1	139	48.9	Ref		
**Number of current ASMs**							
One AED ASMs	100	44.2	126	55.8	Ref		
>=2 AED ASMs	90	69.8	39	30.2	1.58	1.31, 1.90	**<0.001**

**Table 6:** Multivariate Logistic Regression of variables with Seizure control on treatment in Tikur Anbessa Hospital, Pediatric neurology follow up clinic, 2018

Variables	Multivariate Analysis		
	Odds Ratio	95% CI	p value
**Comorbid neurological condition**			
Yes	1.29	1.08, 1.54	**0.005**
No	Ref		
**Number of current Antiseizure drugs**			
One ASMs	Ref		
>=2 ASMs	1.60	1.34, 1.92	**<0.001**

## Discussion

### Multiple Antiseizure medications use

The study found that 36.3% children with epilepsy in the clinic were on multiple Antiseizure medications. The frequency of multiple antiseizure medications use in this study is similar to that described in previous Ugandan study but higher than the 19.5 % of adult epileptic patients in university of Gondar referral hospital study. In most of studies the use of multiple Antiseizure medications was found to be 30 – 40% which is also comparable with this study. 12,16,18

### Pattern of seizure control

In this study almost half (47%) of the participants were seizure free in the past six month which is comparable to study in Yirgalem general hospital southern Ethiopia in which the 51% of the participants achieved seizure control by Antiseizure medications. 17. 60% to 70% of patients will achieve seizure freedom with antiseizure medications. This study result was found to be lower than the above finding 5. More than 90 percent of patient with epilepsy in the clinic had been evaluated by pediatric residents in their first visit. Lack of adequate knowledge in classification of seizure can lead to inappropriate selection of Antiseizure medications that resulted in poor seizure control.

### Factors associated with use of multiple Antiseizure medication therapy

In this study we found that age of onset of seizure in majority of children was in greater than one year of age. In our study children who had seizure in the past six month had two times more likely to be on polytherapy as compared with children with no seizure in the past six month. This is comparable with Ugandan study. Status epilepticus was frequently occurred in children with monotherapy as compared to those children with polytherapy. There is no difference seen between type of epilepsy, the presence of comorbid illness and multiple Antiseizure medications use.

### Antiseizure medications combination

In this study the most common drug combination was sodium valproate and phenobarbital, followed by phenytoin and phenobarbitone. The addition of valproate to phenobarbital may increase plasma concentration of phenobarbital with concomitant toxicity. Phenytoin, an enzyme inducer, significantly affects plasma levels of other drugs. It often raises the plasma concentration of phenobarbital. Carefully monitoring of plasma level of these drugs and are important when using in combination. 8

In our study 91 (25.6%) children had monthly seizure, 59 (11%) had weekly seizure and 24(6.8%) of children had daily seizures. Proper evaluation of these children for other option on epilepsy management including epilepsy surgery is important.

### Factors associated with poor seizure control

We found that children managed with multiple Antiseizure medications were 1.6 times more likely to have poor seizure control as compared to those using monotherapy. This finding is comparable with other African studies. 18. The finding may have been due to those children introduced polytherapy were those who failed to achieve seizure control in monotherapy. Children with comorbid illness have poor seizure control as compared with no other comorbid illness which might be due to children with developmental delay and cerebral palsy could have underlying brain structural abnormality.

Monitoring of serum antiepileptic drug levels can be very useful for establishing the initial dosing schedule. In our study Serum level measurement was determined in only 46 (13.4%) of children particularly those with poorly controlled seizure. Among these children 32 (69.6%) of children serum level was in therapeutic range.

In conclusion, one third of children with epilepsy attending the pediatric neurology clinics at Tikur Anbessa Hospital are being managed with multiple Antiseizure medications.

Many children continued to have seizures despite use of multiple Antiseizure medications. This clearly shows that there is a greater need for better diagnosis to find underlying cause, need for other antiseizure medications or precision therapies, epilepsy surgery and ketogenic diet as anti-epilepsy treatment options. Strategies are also required to support availability of newer Antiseizure medications that can help minimize drug-drug interaction in combination use. Training health workers on Epilepsy treatment and increased access to monitoring drug levels may improve the rational use Antiseizure medications and seizure control. Further studies are required on detail analysis of factors associated with poor seizure control.

## Figures and Tables

**Table 2:** Clinical characteristic of study population, in Tikur Anbessa Hospital, Pediatric neurology follow up clinic, 2018

Characteristic		Number	Percent
Age onset of seizure	< 1 year	115	32.4
	1 - 10year	224	63.1
	10 - 18 year	16	4.5
Duration of epilepsy	6 months to 2 years	78	22
	>2 year	277	78
Type of seizure	Foal aware	19	5.4
	Focal impaired awareness	16	4.5
	Focal to bilateral	26	7.3
	Generalized	294	82.8
	Epilepsy syndrome	13	3.7
Prior history of status epilepticus	Yes	46	13
	No	309	87

**Table 4:** Multivariate Logistic Regression of variables with number of current Anti seizure medication, in Tikur Anbessa Hospital, Pediatric neurology follow up clinic, 2018

Variables	Multivariate Analysis		
	COR	95% CI	p value
**Patient has history of status epilepticus**			
Yes	Ref		
No	0.38	0.19, 0.75	0.005
**Seizure control on treatment**			
No seizure in the past six month	Ref		
Seizure in the past six month	2.03	1.49, 2.76	<0.001
